# Prevalence of Primary, Prominent, and Predominant Negative Symptoms of Schizophrenia in Routine Inpatient Psychiatric Care

**DOI:** 10.3390/medicina62010030

**Published:** 2025-12-23

**Authors:** Jonas Montvidas, Edas Kačerginskis, Paulina Petraitytė, Eimantas Zauka, Virginija Adomaitienė

**Affiliations:** Psychiatry Department, Lithuanian University of Health Sciences, 44307 Kaunas, Lithuania

**Keywords:** schizophrenia, negative symptoms prevalence, brief negative symptoms scale, self-evaluation of negative symptoms scale, prominent negative symptoms, predominant negative symptoms, primary negative symptoms, core negative symptoms

## Abstract

*Background and Objectives*: Prevalence data of different classes of negative symptoms in an inpatient setting are scarce. Most of the data are collected in clinical trials under strict inclusion criteria, using older psychometric scales with known content validity flaws. The objectives of this study were to report the prevalence of different classes of negative symptoms in a naturalistic inpatient setting using both older and newer psychometric scales and to determine whether second-generation psychometric scales were not inferior to older scales in detecting negative symptoms. *Materials and Methods*: Negative symptoms were evaluated using older-generation (Positive and Negative Symptoms Scale, PANSS) and second-generation (Brief Negative Symptoms Scale, BNSS, and Self-Evaluation of Negative Symptoms Scale, SNS) measures. Primary, prominent, core, and predominant negative symptoms were calculated according to various descriptions used in other studies. *Results*: A total of 323 participants were included in this study. The mean score of the PANSS negative symptoms subscores was 25.33 (SD = 6.86, 95% CI 24.57–26.09), the mean total BNSS score was 36.48 (SD = 15.93, 95% CI 34.74–38.23), and the mean SNS total score was 18,9 (SD = 8.99, 95% CI 17.91–19.89). The prevalence of primary negative symptoms was similar in all groups. PANSS and BNSS categorized 71% of the sample as having prominent negative symptoms. The average prevalence of predominant negative symptoms, as measured by PANSS and BNSS, was 9.6%, whereas SNS reported 10.5%. *Conclusions*: Negative symptoms were highly prevalent in our inpatient sample. Most of our participants had primary and prominent negative symptoms; however, only a small fraction met the criteria for predominant negative symptoms. Prevalence data were similar between PANSS, BNSS, and SNS, showing that BNSS and SNS are not inferior to PANSS.

## 1. Introduction

The classification of negative symptoms is complex [1,2,3]. The definition of negative symptoms remained unclear until 2005, when it was agreed that negative symptoms consisted of at least five core symptoms: alogia, abulia, anhedonia, avolition, and blunted affect [4]. Moreover, it was agreed that negative symptoms can be further classified into primary and secondary symptoms [4]. Primary negative symptoms are a neurobiologically and clinically distinct part of schizophrenia. Secondary negative symptoms are symptoms that resemble primary negative symptoms but are caused by positive, depressive, extrapyramidal, or other types of symptoms [5].

Negative symptoms are categorized as core, prominent, predominant, or persistent, leading to a complex terminology [6,7]. Prominent negative symptoms are at least moderately severe and more evident than positive symptoms. Predominant symptoms are moderately severe, stand out over positive symptoms, and occur without significant depressive or extrapyramidal features. Core symptoms align with established consensus groups, while persistent symptoms remain stable and marked for a set period—commonly six months [8]. However, there is no clear consensus on how to measure these symptom types, which contributes to variability across studies that use “prominent” or “predominant” criteria [9].

Negative symptoms are common in patients with schizophrenia, but the literature on their prevalence in a routine inpatient setting remains inconclusive. Most epidemiological data are collected during clinical trials, using tightly controlled samples that consist primarily of stable outpatients. Approximately 50% to 95% of patients with schizophrenia from clinically stable outpatient settings have at least one negative symptom, and around 40% have at least two [6]. Data on the prevalence of primary vs. secondary negative symptoms are even scarcer and show that approximately 13% of patients with schizophrenia have primary negative symptoms [10,11]. Also, we did not find comprehensive studies looking into the prevalence of negative symptoms specifically in an inpatient setting.

Furthermore, data on the prevalence of different classes of negative symptoms are even rarer and less reliable because the definitions of prominent and prevalent negative symptoms vary across studies, making it difficult to combine and compare data [12,13,14]. We did not find studies looking into the prevalence of different classes of negative symptoms in an inpatient setting. Moreover, it is important to note that most epidemiological data are collected using older-generation scales with known content validity flaws, which may lead to discrepancies in prevalence estimates [9].

Psychometric scales for evaluating negative symptoms can be divided into first- and second-generation tools, depending on whether they align with the consensus definition of negative symptoms [15]. First-generation psychometric scales do not align with current descriptions of negative symptoms, resulting in flaws in content validity. Therefore, the scores of these subscales may not reflect only the severity of negative symptoms but also a hybrid factor of cognitive deficits and negative symptoms, which might make the prevalence and severity data somewhat imprecise [9].

Second-generation psychometric scales for the evaluation of negative symptoms adhere to the consensus on negative symptoms. The most prominent examples of this group of psychometric tools are the Brief Negative Symptoms Scale and Self-evaluation of Negative Symptoms Scale [16,17]. According to current guidelines for negative symptom evaluation, it is recommended to use an observer-rated second-generation psychometric scale, supplemented by a self-rated scale, to assess negative symptoms. Using these scales, we should be able to collect the most accurate epidemiological data [9]. However, despite this recommendation, most epidemiological data are collected using PANSS in a strictly controlled clinical trial setting.

Therefore, the primary aim of this study was to report the prevalence of various classes of negative symptoms in routine psychiatric inpatient samples, using both first- and second-generation psychometric tools. The secondary aim was to check whether the second-generation scales were non-inferior to the most widely used first-generation scales.

## 2. Methods and Materials

We included patients from routine inpatient psychiatric care diagnosed with schizophrenia and treated in a tertiary university hospital in Kaunas, Lithuania, between January 2024 and August 2025. The inclusion criteria were a diagnosis of schizophrenia, age between 18 and 65 years, and willingness to participate in the study. The exclusion criteria were comorbidity with intellectual disability, active substance abuse disorders, and/or severe somatic diseases. Patients who met the inclusion criteria and did not meet the exclusion criteria were asked to participate in the study. Patients who agreed to participate in the study and signed an informed consent form were included. The study design was cross-sectional, and no follow-up visits were planned.

Our research team consisted of four researchers, who were trained to use the Brief Negative Symptoms Scale (BNSS), the Calgary Depression Scale for Schizophrenia (CDSS), the Self-evaluation of Negative Symptoms Scale (SNS), and the Positive and Negative Symptoms Scale (PANSS). One researcher who was certified in applying and scoring PANSS collected demographic data and administered the PANSS, while the other three administered the BNSS, SNS, and CDSS. Researchers were instructed in using BNSS and CDSS while SNS needed no formal training. All assessments were conducted on a span of two days on the last days of the inpatient treatment.

The BNSS is a semi-structured interview consisting of 13 items evaluating five domains of negative symptoms and a sixth domain: lack of normal distress. Each item was rated on a scale from 0 (normal) to 6 (extremely severe). The scores for each subscale and the total score can be calculated by summing the item scores [18,19,20]. Core negative symptoms were considered when the BNSS score of ≥3 (moderate) was present on at least one item of any of the five BNSS subscales [20]. To calculate the prevalence of primary and secondary negative symptoms using the BNSS, we included patients with at least one core negative symptom, then controlled for pronounced depressive symptoms (CDSS > 6) and positive symptoms (PANSS positive symptoms subscore (PANSS PS) > 19). Negative symptoms were considered to be prominent if there were either three subscales with at least one item with a score of ≥3 (moderate) (BNSS3) or two subscales with at least one item with a score of ≥4 (moderately severe) (BNSS4). If the CDSS score was <6 and the PANSS PS score was <19, prominent negative symptoms were considered predominant.

SNS is a self-evaluation scale comprising 20 items that assess all five negative symptoms. A patient is asked to indicate whether they agree (2 points), neither agree nor disagree (1 point), or do not agree (0 points) with each statement. Scores of subscales and the total score were calculated by summing the scores of each item [17,21]. Negative symptoms were considered prominent if at least three subscales had at least a single item with a score of ≥2. If the CDSS score was <6 and the PANSS PS score was <19, prominent negative symptoms were considered predominant.

The PANSS is a first-generation semi-structured interview consisting of 30 items, each rated on a 7-point Likert scale from one (absent) to seven (extreme). The PANSS originally had three subscores: positive symptoms (items P1–P7), negative symptoms (items N1–N7), and general psychopathology (items G1–G16) [22]. However, it is well documented that the items in the negative symptoms subscale do not align with the NIMH consensus definition of negative symptoms. It contains items attributable to cognitive deficits such as “difficulty in abstract thinking” (N5) and “stereotyped thinking” (N7). MARDER factors were developed to improve the content validity of the PANSS and are now often used to give numerical meaning to the severity of schizophrenia symptoms (Table 1) [23,24,25,26].

In order to calculate core negative symptoms using the PANSS, a score of ≥4 (moderate) was required for the PANSS items: N1 (blunted affect), N2 (emotional withdrawal), N3 (poor rapport), N4 (passive social withdrawal), and N6 (lack of spontaneity). We did not find a clear description of which PANSS items should be used or of their cut-off score for inclusion as primary negative symptoms. Therefore, we used different descriptions we found in the literature. We included items with a score of ≥ 4 on items in the PANSS negative symptoms subscale (PANSS NS) and MARDER negative symptoms factor (MARDER NF), as well as items belonging to core negative symptoms (PANSS CS). We then controlled for depressive symptoms (CDSS > 6) and positive symptoms (PANSS PS > 19). However, we did not control for extrapyramidal symptoms in either PANSS or BNSS because of a lack of available psychometric tools for this in the Lithuanian language.

To evaluate prominent and predominant negative symptoms, we used the different descriptions mentioned in the European Psychiatric Association (EPA) guidelines for negative symptoms [9]. Prominent negative symptoms were described as at least three moderate (score ≥ 4) (PANSS4) items or at least two moderately severe (score ≥ 5) (PANSS5) items on the negative symptom subscale of PANSS. Predominant negative symptoms are prominent negative symptoms controlled for PANSS PS to be <19 and depressive symptoms lower than the defined threshold of the scale (CDSS < 6).

To have more comparable results we also used the definitions suggested by Harvey et al. in a recent study on the prevalence of prominent and predominant negative symptoms [27]. There are various descriptions of prominent and predominant negative symptoms in the literature and the ones used by Harvey et al. were taken from two recent large-sample influential pharmacological studies focusing on the treatment negative symptoms. Prominent negative symptoms were considered to be when PANSS NS or MARDER NF was more than or equal to either 20 or 24. Predominant negative symptoms were considered when prominent symptoms were also controlled for agitation-based factors, broad-psychosis factors, and/or core-psychosis factors, where certain PANSS items had to have a score of less than four (Table 2). These prominent symptoms were considered predominant when depressive symptoms (CDSS > 6) were also not present.

We used the CDSS to evaluate depressive symptoms. It is a semi-structured interview consisting of 9 items, each rated from 0 (absent) to 3 (severe). The total CDSS score was calculated by summing the scores for each item. The CDSS is a psychometric tool used to evaluate depressive symptoms specifically in patients with schizophrenia. A total score of six or more signifies clinically meaningful depressive symptomatology [28,29].

SPSS version 30.0 was used to perform statistical analysis. Demographic data were calculated using descriptive statistics. All variables were checked for normality using the Shapiro–Wilk criteria. The means for each subscale and the total score were calculated. Either the Student’s *t*-test or the Mann–Whitney U test was used to assess differences between the groups of primary and secondary, prominent and non-prominent, and predominant and non-predominant negative symptoms to determine how each grouping separated patients by the severity of their negative symptoms.

Bioethics approval No. BE-2-70 was received from the Kaunas Regional Biomedical Research Ethics Committee on the 8th of December 2023. Informed consent for participation was obtained from all subjects involved in the study.

## 3. Results

### 3.1. Demographic Data

A total of 323 participants were included in this study. The sample was homogeneous with respect to sex (Chi^2^ = 0.29; *p* = 0.87): 50.5% (n = 158) were male and 49.5% (n = 155) were female. The other demographic data are shown in Table 3. None of the demographic variables were normally distributed according to the Shapiro–Wilk test, and none differed between sexes (*p* > 0.05). The mean CDSS score was 5.73 (SD, 5.03). Only a third of the sample (36.2%, n = 117) had a CDSS score below 6.

### 3.2. Prevalence and Severity of Negative Symptoms

The mean score of the PANSS NS was 25.33 (SD = 6.86, 95% CI 24.57–26.09) and the mean MARDER NF was 25.7 (SD 7.98, 95% CI 24.82–26.57). Mean PANSS NS was higher than the mean PANSS (20.9, SD = 6.68, 95% CI 20.17–21.64); however, mean MARDER NF was almost equal to mean MARDER PF (23.19, SD = 7.98, 95% CI 22.34–24.05). Negative symptoms with the highest scores were N1: Blunted affect (4.53, SD 1.44, 95% CI 4.37–4.68), N4: Passive social withdrawal (4.24, SD 1.56, 95% CI 4.07–4.42), and N2: Emotional withdrawal (4.14, SD 1.31, 95% CI 4–4.29). The average scores of all PANSS items, subscales, and MARDER factors are shown in Table 4. More than half of the sample (59.1%, n = 191) had a PANSS PS score < 19.

The mean total BNSS score was 36.48 (SD = 15.93; 95% CI: 34.74–38.23). Items belonging to avolition and blunted affect subscores had the highest mean scores: behavior (3.11, SD = 1.52; 95% CI 2.95–3.26), internal experience (3.11, SD 1.62; 95% CI 2.93–3.29), facial expression (3.28, SD = 1.5; 95% CI 3.12–3.45), expressive gestures (3.28, SD 1.59, 95% CI 3.1–3.45). The mean scores of all the items and subscales of the BNSS are shown in Table 5.

The mean total score for SNS was 18.9 (SD = 8.99; 95% CI: 17.91–19.89). The avolition subscale had the highest mean subscore of 4.84 (SD = 2.98, 95% CI 4.51–5.16), followed by the social isolation subscale (3.8, SD = 2.15, 95% CI 3.57–4.04), alogia subscale (3.74, SD = 2.38, 95% CI 3.48–4), blunted emotion subscale 3.52, SD = 2.01, 95% CI 3.3–3.74), and anhedonia subscale (3, SD = 2.31, 95% CI 2.74–3.25). Most of the participants had a total SNS score of 7 or higher (92.9%, n = 300).

Core negative symptoms were similarly prevalent when controlled by BNSS, SNS, or PANSS. The prevalence of core negative symptoms, as measured by the PANSS and BNSS, is shown in Table 6.

### 3.3. Primary and Secondary Negative Symptoms

Secondary negative symptoms were more prevalent than primary negative symptoms. The prevalence of primary negative symptoms was similar in all groups: PANSS Core group (19.8%, n = 64), PANSS NS group (19.5%, n = 63), PANSS NF group (19.5%, n = 63), and BNSS group (17%, n = 55).

When comparing the severity of negative symptom scores between the primary and secondary negative symptom groups, the primary negative symptom group had higher scores across all negative symptom scales (BNSS, PANSS Core, PANSS NS, and PANSS NF). Still, the differences were primarily non-significant, except for the BNSS alogia subscore.

### 3.4. Prominent Negative Symptoms

Both BNSS3 (68.1%, n = 220) and PANSS4 (71.8%, n = 232) groups showed similar prevalence. However, the BNSS4 group (58.8%, n = 190) had a higher prevalence compared to the PANSS5 group (48.3%, n = 156). SNS had a similar prevalence (56.7%, n = 183) in the BNSS4 and PANSS5 groups. The PANSS20 (80.2%, n = 259), PANSS24 (62.7%, n = 201), MARDER20 (77.1%, n = 249), and MARDER24 (60.1%, n = 194) showed similar results compared to PANSS4-5 and BNSS3-4.

All prominent NS groups were able to distinguish participants by NS severity. The participants in the prominent NS group had higher NS scores. However, only PANSS4 and PANSS5 were able to separate patients with higher and lower NS scores, with statistical significance (*p* < 0.05) in all NS scores and subscores. BNSS3 performed similarly; however, there was no statistically significant difference between the prominent and non-prominent negative symptom groups in the PANSS NS and PANSS MF subscores. All other groupings (PANSS20, PANSS24, MARDER20, MARDER24, and BNSS4) failed to distinguish patients with higher NS scores at a statistically significant level.

### 3.5. Predominant Negative Symptoms

Only a small fraction of the sample had predominant negative symptoms when evaluated with either BNSS, PANSS or SNS. The prevalence of the predominant negative symptoms is shown in Table 7. The average prevalence of predominant negative symptoms in the groups was 9.6% (n = 31).

All predominant negative symptom groupings separated patients according to the severity of their negative symptoms. Patients with higher negative symptom scores were predominantly in the negative symptom group. However, none of the groups could separate patients according to the severity of their negative symptoms with statistical significance. PANSS_24_BrP and MARDER_24_Ag were able to separate patients with higher scores with statistical significance in all groups, except SNS scores.

## 4. Discussion

Our study found a high prevalence of pronounced negative symptoms in an inpatient sample of schizophrenia patients. About 86–87% of the sample had at least one negative symptom scoring “moderate” on either PANSS, SNS, or BNSS. Our results on the prevalence of negative symptoms differ from those of other studies that included only patients without recent exacerbations. For example, Galderisi et al. used data from the EUFEST study and found that 54% of the sample had at least one “moderate” negative symptom, as assessed by the PANSS [30]. Mucci et al. excluded patients who had an exacerbation or treatment modifications in the last 3 months and found that 59% had at least one “moderate” negative symptom, as evaluated by the BNSS [20]. We believe that we found higher prevalence of negative symptoms because of the nature of our sample. Patients in an inpatient setting usually experience an exacerbation of positive symptoms of schizophrenia, which could increase the prevalence of total negative symptoms by an increased prevalence of secondary negative symptoms. In contrast, in the RCTs, the sample usually consists of relatively stable, strictly controlled patients in an outpatient setting having far less positive symptoms and possibly less secondary negative symptoms.

We used two second-generation scales, BNSS and SNS, which had many more items dedicated to negative symptom evaluation and were faster, easier to use, and at least not inferior, and possibly even better, at negative symptom detection than PANSS. Similar to the findings of Mucci et al., we found that BNSS was more sensitive in identifying negative symptoms, with at least moderate sensitivity (68.1%). In contrast, both PANSS-NS and MARDER-NF showed 55.1% sensitivity [20]. This difference changed when we used criteria for PANSS core symptoms—both BNSS and PANSS identified 68.1 and 64.1% of samples as having at least three core negative symptoms of at least “moderate” sensitivity. On the other hand, BNSS was more sensitive than PANSS in identifying patients with all five core negative symptoms at a severity of at least moderate; BNSS identified 39.6% as such, whereas PANSS identified 22.3%. SNS identified a similar number of participants with at least one core negative symptom of moderate severity (86.1%, n = 277), but had lower sensitivity than both PANSS and BNSS for identifying patients with all five pronounced core negative symptoms (19.2%, n = 62). Similar to Dollfus et al., we found that most of the sample had passed the threshold of SNS total score of 7 (92.9%, n = 300) when not controlled for depression and positive symptoms [21]. Furthermore, BNSS and SNS were easier to use and faster, making them scales of choice for negative symptoms evaluation in an inpatient setting.

Prominent negative symptoms were highly prevalent. The highest prevalence was observed with the PANSS20 criteria (80.2%, n = 259), and the lowest with the PANSS4 criteria (48.3%, n = 156), indicating the highest and lowest thresholds. Our findings differ from those of Harvey et al., who found that only 20% of their sample met the PANSS-20 criteria for prominent negative symptoms [27]. We think this is related to different inclusion criteria; our sample was a naturalistic inpatient sample with a significant prevalence of secondary negative symptoms, and Harvey et al. used data from two randomized controlled trials with strict inclusion criteria.

Regarding the prevalence of prominent negative symptoms according to PANSS4 and PANSS5 criteria, we found a prevalence similar to that reported by Stauffer et al. and Rabinowitz et al., who found 65.3% and 62.3%, respectively [14,31]. Mucci et al. found that 38.9% of their sample had prominent negative symptoms (at least three subscales with at least one item with a score of ≥3), compared to 68.1% in our sample, again showing differences in negative symptom severity between outpatient and inpatient settings [20].

The prevalence of predominant negative symptoms varied across scales and criteria, with SNS and BNSS identifying more predominant cases than PANSS. Comparing findings from BNSS, Mucci et al. found predominant negative symptoms in 21.3% of their sample, whereas we found 13% [20,21]. We think this difference might be because Mucci et al. used different criteria to control for positive symptoms: PANSS PS score < 19, whereas Mucci et al. used positive (P1, P3, P5, P6, G9) and disorganization (P2) scores < 4. We found a similar prevalence of predominant negative symptoms according to the PANSS to that reported by Harvey et al., with the only PANSS criterion reaching over 10% prevalence being PANSS20Ag [27].

To the best of our knowledge, this is the first study to apply SNS to distinguish predominant negative symptoms. It was as sensitive in discerning predominant negative symptoms as BNSS or PANSS. In our opinion it is important to mention that SNS has a much faster completion time (3–5 minutes) which makes it a good bedside screening tool. In a previous study by Dollfus et al., an SNS score of more than 7 was associated with an 87.8% probability that negative symptoms are primary, possibly predominant. In our sample, 92.9% had an SNS score of 7 or higher [32]. However, further research to test if SNS is truly more sensitive in discerning predominant negative symptoms compared to other psychometric scales is needed. Our study has some limitations. First, we did not include a measurement of extrapyramidal symptoms when distinguishing between primary and secondary negative symptoms or predominant symptoms. This increased the risk of inflated and less accurate prevalence of primary and predominant negative symptoms. Another drawback is that we did not collect data on psychopharmacotherapy, which might have influenced the prevalence of secondary negative symptoms. Also, we did not include patients with severe somatic illness or active substance abuse, which reduces the generalizability of our results to a broader population of patients with dual diagnosis of schizophrenia and severe somatic diseases or substance abuse. Finally, we would have conducted a follow-up evaluation of the same patient sample to estimate the prevalence of persistent negative symptoms.

Finally, our results add to the general pool of knowledge on the prevalence of negative symptoms in an inpatient setting and of different classes of negative symptoms, and underscore the need for consensus on criteria for primary, prominent, and predominant negative symptoms. We found different prevalence estimates for these classes of negative symptoms across different psychometric scales and criteria used in other studies. Negative symptoms are recognized as an official treatment target by the European Medicines Agency, and the lack of a unified description of their different types might hamper progress toward finding a way to treat them. Therefore, our findings warrant further research and a need for a consensus.

## 5. Conclusions

Negative symptoms were highly prevalent in an inpatient setting. Secondary and prominent negative symptoms were more prevalent; however, only a small fraction of our sample met the criteria for predominant symptoms. The second-generation psychometric scales were noninferior to the PANSS in assessing the prevalence of negative symptoms and distinguishing between classes of negative symptoms. Given their ease of use and noninferiority to PANSS, we believe that BNSS and SNS are the scales of choice for an inpatient setting.

## Figures and Tables

**Table 1:** MARDER positive and negative factors.

MARDER Facto	PANSS Items and Item Titles
Positive factor	P1 delusions, P3 hallucinatory behavior, P6 Suspiciousness/persecution, G9 Unusual thought content
Negative factor	N1 Blunted affect, N2 Emotional withdrawal, N3 Poor rapport, N4 Passive social withdrawal, N6 Lack of spontaneity in flow of conversation, G7 Motor retardation

**Table 2:** Non-predominant negative symptoms exclusion criteria.

Factor to Exclude Non-Predominant Negative Symptoms	PANSS Items < 4
Agitation-based factor	P-4 (Excitement); P-7 (Hostility); P-6 (Suspiciousness); G-8 (Uncooperativeness); G-14 (Poor impulse control)
Broad-psychosis factor	P1 (Delusions); P2 (Conceptual Disorganization); P3 (Hallucinations); P5 (Grandiosity); P6 (Suspiciousness); G9 (Unusual Thought Content)
Core-psychosis factor	P1 (Delusions); P3 (Hallucinations); P6 (Suspiciousness); G9 (Unusual Thought Content)

**Table 3:** Demographic data.

Demographic Variable	Mean Total (SD)	Mean Female (SD)	Mean Male (SD)	*p* Value
Age (years)	36.68 (13.14)	39.28 (14.33)	33.93 (11.21)	0.001
Weight (kg)	79.65 (19.5)	74.82 (19.35)	83.9 (18.5)	<0.001
Years of education	13.95 (2.81)	14.38 (3.1)	13.5 (2.42)	0.008
Years of disease	12.61 (11.62)	14.48 (12.73)	10.75 (10)	0.006

**Table 4:** PANSS negative items, PANSS subscores, and MARDER factors.

PANSS Item	Mean Score (SD)	95% CI	Score ≥ 4; %
N1 (Blunted affect)	4.53 (1.44)	4.37–4.68	78
N2 (Emotional withdrawal)	4.14 (1.31)	4–4.29	72.8
N3 (Poor rapport)	3.04 (1.31)	2.9–3.18	33.1
N4 (Passive social withdrawal)	4.24 (1.56)	4.07–4.42	65.6
N5 (Difficulty with abstract thinking)	3.52 (1.48)	3.36–3.68	48
N6 (Lack of spontaneity and flow of conversation)	3.72 (2.09)	3.49–3.95	42.7
N7 (Stereotyped thinking)	2.67 (1.23)	2.54–2.81	24.5
**PANSS scores and MARDER factors**			
Positive subscore	20.9 (6.68)	20.17–21.64	-
Negative subscore	25.87 (7.51)	25.08–26.65	-
General subscore	44.09 (6.65)	43.15–45.04	-
MARDER Depression/anxiety factor	10.79 (3.22)	10.44–11.14	-
MARDER Uncontrolled hostility/excitement factor	8.96 (3.95)	8.53–9.39	-
MARDER Disorganized thoughts factor	22.22 (5.7)	21.6–22.85	-
MARDER Positive symptoms factor	23.19 (7.82)	22.34–24.05	-
MARDER Negative symptoms factor	25.7 (7.98)	24.82–26.57	-
MARDER Total score	90.86 (17.92)	88.9–92.83	-

PANSS–Positive and Negative Symptom Scale; N1–N7—items of the negative symptom subscale of the Positive and Negative Symptom Scale; EPA-4, at least three items with a score of ≥4 on the negative symptoms subscale; MARDER—the MARDER factors of the Positive and Negative Symptoms Scale; SD—standard deviation; CI—confidence interval.

**Table 5:** BNSS mean scores.

BNSS Item/Subscore	Mean Score (SD)	95% CI	Score ≥ 4; %
**Anhedonia subscale**			
1. Intensity of pleasure during activities	2.69 (1.53)	2.52–2.85	52.9
2. Frequency of pleasurable activities	3.06 (1.49)	2.9–3.23	62.2
3. Intensity of expected pleasure from future activities	2.72 (1.7)	2.53–2.9	55.1
Anhedonia subscore	8.47 (4.38)	7.99–8.95	-
**Asociality subscale**			
4. Lack of normal distress	2.08 (1.52)	1.91–2.25	40.2
5. Asociality: behavior	2.64 (1.52)	2.47–2.81	52.6
6. Asociality: internal experience	2.52 (1.15)	2.35–2.68	46.4
Asociality subscore	5.15 (2.86)	4.84–5.47	-
**Avolition subscale**			
7. Avolition: behavior	3.11 (1.5)	2.95–3.26	64.4
8. Avolition: internal experience	3.11 (1.62)	2.93–3.29	63.2
Avolition subscore	6.22 (2.88)	5.9–6.33	-
**Blunted affect subscale**			
9. Facial expression	3.28 (1.5)	3.12–3.45	69
10. Vocal expression	3.03 (1.69)	2.84–3.21	62.8
11. Expressive gestures	3.28 (1.5)	3.1–3.45	67.8
Blunted affect subscore	9.59 (4.45)	9.1–10.08	-
**Alogia subscale**			
12. Quality of speech	2.26 (1.55)	2.09–2.43	42.1
13. Spontaneous elaboration	2.56 (1.7)	2.38–2.75	50.8
Alogia subscore	4.82 (3.17)	4.48–5.17	-
Total score	36.48 (15.93)	34.74–38.23	-

BNSS—Brief Negative Symptoms Scale; SD—standard deviation; CI—confidence interval.

**Table 6:** Prevalence of core negative symptoms.

Number of Core Symptoms	BNSS		PANSS		SNS	
	N	%	N	%	N	%
0	41	12.7	40	12.4	45	13.9
1	30	9.3	34	10.5	44	13.6
2	32	9.9	42	13	51	15.8
3	39	12.1	71	22	61	18.9
4	53	16.4	64	19.8	60	18.6
5	128	39,6	72	22.3	62	19.2

BNSS—Brief Negative Symptoms Scale; PANSS—Positive and Negative Symptoms Scale; SNS—Self-evaluation of Negative Symptoms Scale.

**Table 7:** Prevalence of predominant negative symptoms.

Type of Predominant Negative Symptoms	Predominant Negative Symptoms % (*n*)
EPA-4	14.6 (47)
EPA-5	9.3 (30)
PANSS20-AG	15.2 (49)
PANSS24-AG	11.8 (38)
MARDER20-AG	14.2 (46)
MARDER24-AG	10.5 (34)
PANSS20-BrP	5.9 (19)
PANSS245-BrP	3.4 (11)
MARDER20-BrP	5.6 (18)
MARDER24-BrP	3.7 (12)
PANSS20-CrP	11.1 (36)
PANSS24-CrP	6.8 (22)
MARDER20-CrP	10.2 (33)
MARDER24-CrP	6.8 (22)
BNSS3	13 (42)
BNSS4	11.1 (36)
SNS	10.5 (34)

EPA-4, at least three items with a score of ≥4 on the negative symptoms subscale; EPA-5, at least two items with a score of ≥5 on the negative symptoms subscale; PANSS20, PANSS negative subscore ≥ 20; PANSS24, PANSS negative subscore ≥ 24; MARDER20, MARDER negative symptom factor ≥ 20; MARDER24, MARDER negative symptoms factor ≥ 24; AG, agitation factor; BrP, broad-psychosis factor; CrP, core-psychosis factor; BNSS3, three subscales with an item with a score of ≥3; BNSS4, two subscales with an item with a score of ≥4; n, number of participants; SNS—Self-evaluation of negative symptoms scale.

## Data Availability

Data is unavailable due to privacy or ethical restrictions.

## Abbreviations

The following abbreviations are used in this manuscript:

AG	Agitation-based factor
BNSS	Brief Negative Symptoms Scale
BrP	Broad-Psychosis Factor
CDSS	Calgary Depression Scale for Schizophrenia
CI	Confidence Interval
CrP	Core-Psychosis Factor
EPA	European Psychiatric Association
G	PANSS General Psychopathology subscale
N	PANSS Negative Symptoms subscale
NS	PANSS Negative Symptoms subscore
NF	MARDER Negative Factor
P	PANS Positive symptoms subscale
PANSS	Positive and negative symptoms scale
PF	MARER Positive factor
PS	PANSS Positive symptoms subscore
SD	Standard Deviation
SNS	Self-evaluation of negative symptoms scale
TS	Total score
