# Correction: Montvidas et al. Prevalence of Primary, Prominent, and Predominant Negative Symptoms of Schizophrenia in Routine Inpatient Psychiatric Care. *Medicina* 2026, *62*, 30

**DOI:** 10.3390/medicina62020280

**Published:** 2026-01-29

**Authors:** Jonas Montvidas, Edas Kačerginskis, Paulina Petraitytė, Eimantas Zauka, Virginija Adomaitienė

**Affiliations:** Psychiatry Department, Lithuanian University of Health Sciences, 44307 Kaunas, Lithuania

## Error in Author’s Name

In the original publication [1], there was a mistake in the author’s name.

The updated name and email should be the following:

Edas Kačerginskis, edas.kacerginskis@stud.lsmu.lt (E.K.)

## Error in Table

In Table 7, to reflect the prevalence of predominant negative symptoms evaluated with SNS, we have added a line of data [SNS 10.5 (34)] and the abbreviation SNS. The corrected Table 7 is as follows:

**Table 7:** Prevalence of predominant negative symptoms.

Type of Predominant Negative Symptoms	Predominant Negative Symptoms % (*n*)
EPA-4	14.6 (47)
EPA-5	9.3 (30)
PANSS20-AG	15.2 (49)
PANSS24-AG	11.8 (38)
MARDER20-AG	14.2 (46)
MARDER24-AG	10.5 (34)
PANSS20-BrP	5.9 (19)
PANSS245-BrP	3.4 (11)
MARDER20-BrP	5.6 (18)
MARDER24-BrP	3.7 (12)
PANSS20-CrP	11.1 (36)
PANSS24-CrP	6.8 (22)
MARDER20-CrP	10.2 (33)
MARDER24-CrP	6.8 (22)
BNSS3	13 (42)
BNSS4	11.1 (36)
SNS	10.5 (34)

EPA-4, at least three items with a score of ≥4 on the negative symptoms subscale; EPA-5, at least two items with a score of ≥5 on the negative symptoms subscale; PANSS20, PANSS negative subscore ≥ 20; PANSS24, PANSS negative subscore ≥ 24; MARDER20, MARDER negative symptom factor ≥ 20; MARDER24, MARDER negative symptoms factor ≥ 24; AG, agitation factor; BrP, broad-psychosis factor; CrP, core-psychosis factor; BNSS3, three subscales with an item with a score of ≥3; BNSS4, two subscales with an item with a score of ≥4; *n*, number of participants; SNS, self-evaluation of negative symptom scale.

## Text Correction

Due to the author’s negligence, some texts need to be updated in the Abstract and Sections 3.5 and 4.

In the Abstract, the sentence “The average prevalence of predominant negative symptoms, as measured by PANSS and BNSS, was 9.6%, whereas SNS reported 76.2%” should be updated to the following version:

“The average prevalence of predominant negative symptoms, as measured by PANSS and BNSS, was 9.6%, whereas SNS reported 10.5%”.

In Section 3.5, the sentence “Only a small fraction of the sample had predominant negative symptoms when evaluated with BNSS or PANSS, but the proportion was higher with SNS (76.2%, n = 246)” should be updated to the following version:

“Only a small fraction of the sample had predominant negative symptoms when evaluated with either BNSS, PANSS, or SNS.”

In Section 4, the sentence “The fact that SNS did not identify a higher prevalence of prominent negative symptoms compared to PANSS or BNSS but did identify a much higher prevalence of predominant negative symptoms (76.2%) might indicate that SNS is more sensitive to detecting predominant negative symptoms” should be updated to the following version:

“It was as sensitive in discerning predominant negative symptoms as BNSS or PANSS. In our opinion, it is important to mention that SNS has a much faster completion time (3–5 min) which makes it a good bedside screening tool.”

The authors state that the scientific conclusions are unaffected. This correction was approved by the Academic Editor. The original publication has also been updated.
